# Iron-based magnetic superhalogens with pseudohalogens as ligands: An unbiased structure search

**DOI:** 10.1038/srep45149

**Published:** 2017-03-22

**Authors:** Li Ping Ding, Peng Shao, Cheng Lu, Fang Hui Zhang, Li Ya Wang

**Affiliations:** 1 Department of Optoelectronic Science & Technology, College of Elecrical & Information Engineering, Shanxi University of Science & Technology, Xian, 710021, China; 2 Department of Physics, Shaanxi University of Science & Technology, Xi’an, 710021, China; 3 Department of Physics, Nanyang Normal University, Nanyang, 473061, China

## Abstract

We have performed an unbiased structure search for a series of neutral and anionic Fe*L*_4_ (*L* = BO_2_, CN, NO_2_, NO_3_, OH, CH_3_, NH_2_, BH_4_ and Li_2_H_3_) clusters using the CALYPSO (Crystal structure Analysis by Particle Swarm Optimization) structure search method. To probe the superhalogen properties of neutral and anionic Fe*L*_4_ clusters, we used density-functional theory with the B3LYP functional to examine three factors, including distribution of extra electron, pattern of bonding and the nature of the ligands. Theoretical results show that Fe(BO_2_)_4_, Fe(NO_3_)_4_ and Fe(NO_2_)_4_ can be classified as magnetic superhalogen due to that their electron affinities even exceed those of the constituent ligands. The magnetic moment of Fe atom is almost entirly maintained when it is decorated with various ligands except for neutral and anionic (Li_2_H_3_)_4_. Moreover, the current work is also extended to the salt moieties formed by hyperhalogen/superhalogen anion and Na^+^ ion. It is found that these salts against dissociation into Na + Fe*L*_4_ are thermodynamic stable except for Na[Fe(OH)_4_]. These results provides a wealth of electronic structure information about Fe*L*_4_ magnetic superhalogens and offer insights into the synthesis mechanisms.

The molecules and clusters with electron affinities span the 3.6–14 eV range^1^,^2^ have attracted considerable attention in recent years because their negative ions play an important role in the chemical industry, such as constituents of salts, oxidizing and purifying agents. This class of extraordinary compounds is termed as “superhalogens”^2^, which is a kind of “superatoms”^3^,^4^,^5^, due to the fact that their electron affinities are even larger than that of the chlorine element (3.6 eV)^6^. They were first proposed by Boldyrev and Gutsev in 1981^1^, who introduced a simple formula of *MX*_*K*+1_ to describe one class of superhalogens. Originally, *M* is a main group or transition metal atom, *X* is a halogen atom in this formula. From then on, numerous experimental^7^,^8^,^9^ and theoretical works^10^,^11^ have devoted to search for superhalogens and their anions.

The early studies mainly concentrated on main-group metals decorated with halogens. Along with the growing interest in the design of new superhalogens, later studies have shown that O and H atoms can also be ligands^11^,^12^,^13^. Recently, the pool of superhalogens has been further extended by using pseudohalogens^14^ (e.g. CN^15^, BO_2_^16^) as building blocks. According to the isoelectronic theory, Anusiewicz and co-workers^17^ performed a theoretical search for alternative 9-electron (i.e., isoelectronic with F atom) species that possibly serve as ligands in superhalogen anions. They found that the OH, Li_2_H_3_, and NH_2_ groups might be considered as alternative ligands due to their thermodynamic stabilities and large values of electron binding energy. As for other types of pseudohalogens, *M*(BO_2_)_*n*_ systems (*M* = Au, Cu, Na, Mg, Al, Fe and Mn)^18^,^19^,^20^,^21^,^22^ with BO_2_ superhalogen as ligand, *M*(CN)_*n*_ (*M* = Li, Na, Mg, Be, Ca, B, Al and Au)^15^,^23^,^24^ with CN moiety as building block, as well as various different moieties such as BH_4_ and BF_4_^25^, electrophilic NO_2_^26^, and acidic functional groups NO_3_^27^ have been investigated.

Recently, Wu *et al*.^28^ discovered a new class of magnetic superhalogen *M*_*x*_Cl_2*x*+1_^−^ cluster with molecular composition *x* = 1, 2, 3, 4, …. In *M*_*x*_Cl_2*x*+1_^−^ clusters, the outer 4s^2^ electrons of the Mn atom participate in chemical bonding, thereby leaving the half-filled 3d^5^ shell to carry a magnetic moment of 5 μ_B_. In this case, these magnetic superhalogens with magnetic and superoxidizing properties have the potential to serve as building blocks of magnetic materials. In order to acquire a comprehensive understanding of the magnetic coupling, the isoelectronic analogues of [Mn_2_Cl_5_]^−^ (i.e. cationic [Fe_2_Cl_5_]^+^ and neutral MnFeCl_5_) were investigated by Yin groups in 2012^29^. They found that the Fe_2_Cl_5_^+^ possesses the stronger magnetic coupling, and the degree of spin delocalization is larger than that in Mn_2_Cl_5_^−^. Inspired by these results, we have performed investigations on iron-based magnetic superhalogens with halogen (F, Cl, and Br)^30^ or interhalogen (ClF, ClF_3_, ClF_5_, BrF, BrF_3_, BrF_5_, and BrCl)^31^ as ligand. However, most of the previous works of magnetic superhalogens are concentrated on halogen atoms as ligand. To the best of our knowledge, the investigation performed on the magnetic superhalogen, especially for the pseudohalogens as ligand is scare.

In this study, we explore the possibilities for new class of magnetic superhalogens or hyperhalogens by using Fe as center atom and BO_2_, CN, NO_2_, NO_3_, nine electrons OH, CH_3_, NH_2_, BH_4_ and Li_2_H_3_ as ligands. BO_2_^18^,^19^,^20^,^21^,^22^, CN^23^,^24^, NO_2_^26^ and NO_3_^27^ have been successfully used as ligands to synthesize superhalogens. According to the isoelectronic theory, OH, CH_3_, NH_2_, BH_4_ and Li_2_H_3_, which are 9-electron species (i.e., isoelectronic with F atom), may possibly serve as ligands in superhalogens. Our original motivation for this study is to determine if the electron affinities of these complexes can exceed those of their building blocks or not? If so, these complexes will be termed as superhalogens or hyperhalogens. In addition, do these clusters, which have pseudohalogens as ligand, possess other excellent properties. Equally important is to see if the iron atom continues to carry magnetic moment. Compared with its magnetic moment of isolate atom (4 μ_B_/atom), the values will be decreased or enhanced with ligand decoration? The paper is organized as follows. The computational methodology, along with the technical details regarding the structure search is shown in Sec. II. Results are presented and discussed in Sec. III. Finally, the main conclusions are summarized in Sec. IV.

## Computational Methods

To search for the lowest-energy structures of neutral and anionic Fe*L*_4_ (*L* = BO_2_, CN, NO_2_, NO_3_, OH, CH_3_, NH_2_, BH_4_ and Li_2_H_3_) clusters, a two-step computation procedure is undertaken. Firstly, an unbiased structure search is performed, using the CALYPSO (Crystal structure Analysis by Particle Swarm Optimization) structure search method combined with B3LYP functional^32^,^33^,^34^. This method has successfully predicted the ground state structures for various systems^35^,^36^,^37^,^38^,^39^,^40^. For each neutral and anionic Fe*L*_4_ clusters, we followed 30 generations to achieve convergence of the search. Each generation contains 50 structures, 60% of which are generated by particle swarm optimization (PSO), while the others are generated randomly. Next, among the 1000–1500 isomers, the top fifteen low-lying isomers are collected as candidates for lowest-energy structure. These isomers with energy difference from the low-lying isomers less than 3 eV are further reoptimized by B3LYP/6-311+G* 41,42 theoretical method. This level of theory has been successful in predicting the electron affinities of a large number of systems correctly^13^,^30^,^43^,^44^,^45^. All the quantum chemical calculations are carried out using Gaussian09 program package^46^. In the geometric re-optimization procedure, various possible spin multiplicities are considered to determine the preferred spin state due to the spin polarization. Meanwhile, the vibrational frequency calculations are performed to make sure that the structures correspond to real local minima without imaginary frequency.

To verify the reliability of the computational method and basis sets, we first calculated the electron affinities of these ligands at B3LYP/6-311+G*, B3LYP/6-311+G(2df) and CCSD/6-311+G* levels. By comparing the theoretical values with the experiment data (see Table 1), it is found that the present results based on the B3LYP/6-311+G* method are more dependable^47^,^48^,^49^,^50^,^51^,^52^,^53^.

## Results and Discussion

**Figure 1:**
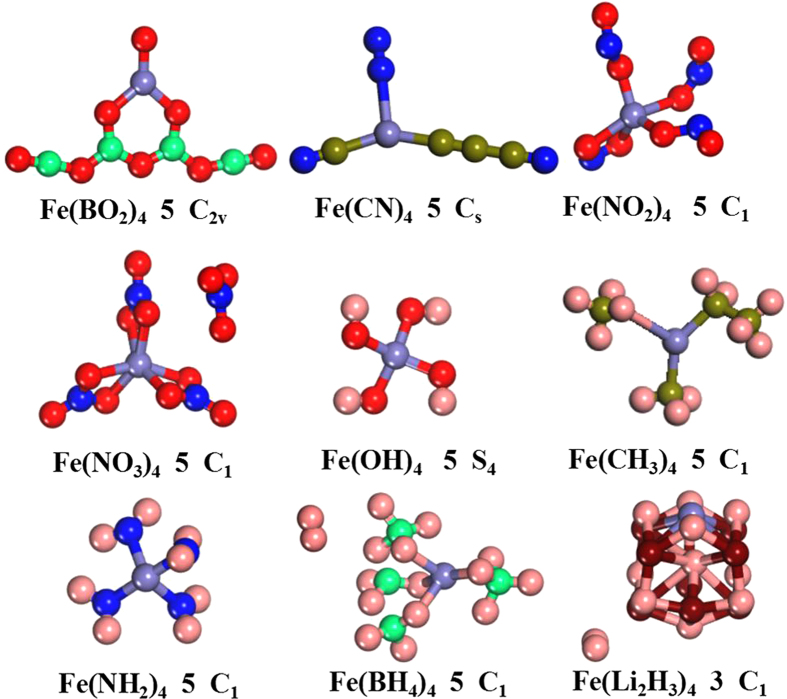
The ground-state structures of neutral clusters Fe*L*_4_ (*L* = BO_2_, CN, NO_2_, NO_3_, OH, CH_3_, NH_2_, BH_4_ and Li_2_H_3_) along with their spin multiplicities and symmetries. The red, springgreen, royalblue, blue, olive, pink and maroon spheres represent the O, B, Fe, N, C, H and Li atoms, respectively.

**Figure 2:**
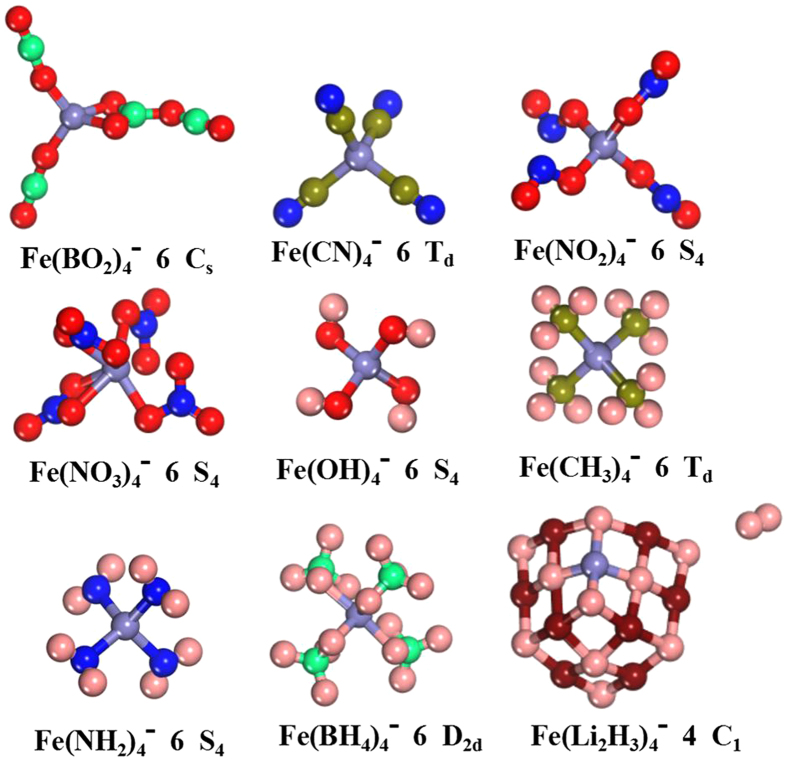
The ground-state structures of anionic clusters Fe*L*_4_ (*L* = BO_2_, CN, NO_2_, NO_3_, OH, CH_3_, NH_2_, BH_4_ and Li_2_H_3_) along with their spin multiplicities and symmetries. The red, springgreen, royalblue, blue, olive, pink and maroon spheres represent the O, B, Fe, N, C, H and Li atoms, respectively.

To probe the magnetic superhalogens/hyperhalogens with pseudohalogen as ligands, we examine the possibility by concentrating on iron atom decorated with superhalogen BO_2_, halogenoid CN, electrophilic NO_2_, acidic functional NO_3_, and nine electrons groups (OH, CH_3_, NH_2_, BH_4_ and Li_2_H_3_, which are isoelectronic with F atom). The low-energy structures of Fe*L*_4_ (*L* = BO_2_, CN, NO_2_, NO_3_, OH, CH_3_, NH_2_, BH_4_ and Li_2_H_3_) clusters can be categorized into two kinds: the ligands are allowed to bind either individually or in group. Here, we only select the lowest-energy structures of neutral and anionic species, respectively. The results are shown in Figs 1 and 2 together with their spin multiplicity, point symmetry and relative energy. The other structure information, including the shortest distance between central Fe and ligand atoms, and the frequencies of the lowest structures are collected in Table S1. The electronic state, the highest occupied molecular orbital (HOMO), the lowest unoccupied molecular orbital (LUMO) and HOMO-LUMO gap of the lowest-energy structures are listed in Table S2. The other two low-lying isomers and their corresponding information are in Figures S1 and S2 of Supplementary Material, respectively. Superhalogen properties are justified afterward through calculated their electron affinities (EAs) and vertical detachment energies (VDEs). Magnetic properties of the ground state structures are discussed subsequently. Taking their EAs, VDEs and magnetism into considerations, some magnetic superhalogens can be found. Finally, the salt moieties consisted of superhalogen anions and Na^+^ ion together with their thermodynamically stabilities are discussed.

### Geometric structures

A wealth of isomers is obtained for neutral and anionic Fe*L*_4_ clusters in the current work. Here, we mainly focus on their lowest energy structures, whereas the other low-lying isomers are only briefly characterized. Now, we begin our discussion with the structures of neutral and anionic Fe(BO_2_)_4_ clusters. The ground state of neutral Fe(BO_2_)_4_ contains a six-membered ring (consisting of a Fe atom, three oxygen atoms, and two boron atoms) with two BO_2_ superhalogen molecules and a terminal oxygen atom. Namely, it has a B_4_O_7_ unit binding to a Fe-O unit. Note that Fe is three-coordinate with O atoms in this ground-state structure. Another isomer, containing two B_2_O_4_ units and Fe atom (Figure S3b), is 0.14 eV higher in energy than the lowest energy isomer. When assembled into Fe(BO_2_)_4_^−^ anion, the lowest energy structure consists of a B_2_O_4_ unit and two separate BO_2_ moieties bonded to the Fe atom (Fig. 2), with the plane of the B_2_O_4_ unit perpendicular to the rest of the cluster. The next higher isomer, in which four BO_2_ moieties are separately bonded to the central Fe atom, is only 0.02 eV above the ground state. Compared with the neutral species, the isomer corresponding to the neutral isomer containing two B_2_O_4_ units is above the ground state by 0.19 eV higher, as shown in Figure S1 of the Supplementary Information. As shown above, the obtained ground-state structures of both neutral and anionic Fe(BO_2_)_4_ clusters are in good agreement with the previous theoretical results^54^. As for the ground-state structure of neutral Fe(CN)_4_, two of the CN moieties perfer to form dimerize (C_2_ and N_2_) while the other two CN are attached to the Fe atom individually. The structure with C_2_N_2_ dimer is less stable than the ground state by only 0.08 eV. However, the ground-state structure of anionic Fe(CN)_4_^−^ is similar to the low-lying isomer b of anionic Fe(BO_2_)_4_^−^ in which each ligand unit is attached separately to the central Fe atom (see Figure S2). The same scenario is also found in both neutral and anionic Fe(NO_2_)_4_ clusters. The only difference is that all four NO_2_ units are bent in the latter. In neutral Fe(NO_3_)_4_ cluster, we can see that the valency +3 of the Fe atom is satisfied by three NO_3_ ligands. The fourth NO_3_ ligand behaves as an individual molecule with the bond length similar to that of the isolated NO_3_ molecule^55^. For anionic Fe(NO_3_)_4_, the deficiency of electron is satisfied through adding an extra electron. It is found that the ground-state structure of Fe(NO_3_)_4_^−^ has S_4_-symmetry with sextet spin multiplicity. The higher-energy isomer (C_1_-symmetry) with quartet spin multiplicity is found to be less stable by relative energy 0.66 eV compared to its ground-state isomer.

We focus our attention on the structures of the systems utilizing ligands containing nine electrons, such as OH, CH_3_, NH_2_, BH_4_ and Li_2_H_3_. To our knowledge, these nine electrons systems decorated Al atom matching the Al*X*_4_^−^ formula have been studied^15^,^17^,^21^,^25^. The results showed that Al*X*_4_ (*X* = OH, CH_3_, NH_2_ and Li_2_H_3_) may be superhalogens. However, transition metal Fe decorated by them has not been investigated so far. As is well known, iron atoms have predominant oxidation states of +2 and +3 and carry a spin magnetic moment of 4 μ_B_/atom, as well as couple ferromagnetically in the bulk. Therefore, it is very interesting to study Fe*L*_4_ (*L* stands for nine electron OH, CH_3_, NH_2_, BH_4_ and Li_2_H_3_). According to our calculations, the lowest-energy structures of both neutral and anionic Fe(OH)_4_ clusters all exhibit high S_4_-symmetry with four hydroxyl OH groups linked to the central metal atom in a tetrahedral manner, as shown in Figs 1 and 2. More importantly, the mutual orientation of OH units allow intramolecular hydrogen bonds among them, which may stabilize this structure additionally. When methyl groups CH_3_ assembled into the anionic Fe(CH_3_)_4_ cluster, the methyl groups preserve their pyramidal structure (the CH_3_ is C_3v_-symmetry pyramidal)^17^. While there are two CH_3_ maintain the pyramidal structure in the ground-state structure of neutral specie. The neutral structure adopted C_1_-symmetry whereas the anion mimics a well-known T_d_-symmetry neopentane structure. As for Fe(NH_2_)_4_ cluster, the neutral lowest-energy structure is similar to that of its corresponding anion in which the four nitrogen atoms connected to the central Fe in a tetrahedral manner. However, the symmetry increase from C_1_ (neutral specie) to S_4_ (anion) due to the symmetrical distribution of each two NH_2_ groups. Interestingly, the structure of NH_2_ moiety in both neutral and anionic Fe(NH_2_)_4_ clusters remain unaltered as its isolated triangle structure^17^ with only minor changes in N-H bond length. Nine electron BH_4_, whose electron affinity (3.17 eV)^25^ is close to that of F, connects to transition metal Fe atom forming Fe(BH_4_)_4_^0/−^ clusters. The lowest-energy structures of the neutral and anionic Fe(BH_4_)_4_ clusters are obviously different from each other completely. The neutral Fe(BH_4_)_4_ possesses C_1_-symmetry with only two of the four BH_4_ moieties having intact structure. Two hydrogen atoms, which separate from the rest two BH_4_ moieties respectively, form H_2_ molecule (Fig. 1a). Isomer b, with two BH_4_ molecules dimerize, is 0.60 eV higher in energy. In both of the two neutral isomers, it is found that three of the BH_4_ moieties bind to Fe with H pointing toward Fe, while the fourth BH_4_ moiety binds to Fe with B pointing toward Fe. On the other hand, the ground state of Fe(BH_4_)_4_^−^ cluster is of D_2d_-symmetry with four H atoms connected to the central Fe in a tetrahedral manner (see Fig. 2). When the Li_2_H_3_ system is used as ligand, it is clearly found that there is a H_2_ molecule dissociation in the neutral and anionic ground-state structures. This H-H bond length (0.745 Å) is almost equal to the value of the isolated H_2_ molecule (0.743 Å). Meanwhile, the neutral ground-state structure is similar to low-lying isomer b of anionic Fe(Li_2_H_3_)^−^. It is worth mentioning that a similar structure to the ground-state structure of Al(Li_2_H_3_)_4_^− ^17 with C_2_-symmetry is found in present study. However, their relative energies are estimated to be higher by 1.15 eV and 1.73 eV with respect to the neutral and anionic ground-state isomers, respectively.

### Superhalogen properties

#### Electron affinity and vertical detachment energy

The electron affinity (EA) and vertical detachment energy (VDE) are always used to examine whether a molecule or group belongs to superhalogen. We have calculated electron affinities (EAs = *E*_optimized neutral_ − *E*_optimized anion_) and vertical detachment energies (VDEs = *E*_neutral at optimized anion geometry_ − *E*_optimized anion_) for anionic clusters Fe*L*_4_ (*L* = BO_2_, CN, NO_2_, NO_3_, OH, CH_3_, NH_2_, BH_4_ and Li_2_H_3_). Furthermore, their adiabatic detachment energies (ADEs) are also calculated. The ADE is given by the energy difference between the ground state structure of anion and that of its structurally similar neutral. If the geometries of neutral clusters do not differ from anions significantly, the calculated ADE is considered to be equal to the EA value. The calculated results are given in Table 2. To ensure the accuracy of our calculations, we have repeated the calculations for EAs, VDEs and ADEs at the coupled-cluster single and double substitutions (including triple excitations) CCSD(T) level of theory^56^. The results are also listed in Table 2. The available theoretical and experimental EA and VDE values of FeF_4_^−^, FeCl_4_^−^ and FeBr_4_^−^ are also collected in Table 2. Unfortunately, there is no available experimental data for these clusters. We hope that our theoretical results for these clusters would provide more information for further experiments.

From Table 2, it can be clearly seen that the electron affinities of Fe(BO_2_)_4_^−^, Fe(NO_2_)_4_^−^ and Fe(NO_3_)_4_^−^ are larger than that of Cl which has the highest electron affinity (3.617 eV)^57^ in the periodic table. The EA of Fe(NO_3_)_4_^−^ (6.303 eV) is even larger than the theoretical value of FeF_4_^−^ (6.091 eV)^30^ as well as the experimental EA values of FeCl_4_^−^ (6.00 ± 0.08 eV)^58^ and FeBr_4_^−^ (6.00 ± 0.08 eV)^58^. Thus, these three clusters can be regarded as superhalogens. As shown in Table 1, we also found that the EAs of these three clusters surpass their ligands BO_2_ (4.353 eV), NO_2_ (2.294 eV) and NO_3_ (4.034 eV), respectively. Due to ligands BO_2_ and NO_3_ have been confirmed to be superhalogens in earlier experiments^53^,^59^, hence the Fe(BO_2_)_4_ and Fe(NO_3_)_4_ even can be classified as hyperhalogens. Compared with the obtained results at CCSD(T) level of theory, the electron affinities based on the B3LYP level seem to be slightly underestimated as shown in Table 1. For example, the EAs of Fe(CN)_4_^−^(3.532 eV) and Fe(OH)_4_^−^(4.248 eV) at CCSD(T) level are larger than the values (3.504 and 3.331 eV) obtained at B3LYP level. So, the Fe(CN)_4_ and Fe(OH)_4_ might also be exploited as superhalogen which needs future experiment to confirm. The other four clusters (Fe(CH_3_)_4_^−^, Fe(NH_2_)_4_^−^, Fe(BH_4_)_4_^−^, Fe(Li_2_H_3_)_4_^−^) have the lower EAs and can’t be regarded as superhalogens. As stated above, the values of EA and ADE are the same when the ground-state geometries of the anion and neutral do not differ much. Then, the difference between EA and ADE is a measure of the structural distortion that anion undergoes after detaching an electron. Table 2 shows that only the structures of neutral Fe(OH)_4_ and Fe(CH_3_)_4_ are almost identical to their corresponding anions.

The vertical electron detachment energies of Fe(OH)_4_^−^, Fe(CH_3_)_4_^−^ and Fe(NH_2_)_4_^−^ anions are calculated to be 4.147 eV, 2.731 eV and 3.191 eV, respectively. These relatively small VDEs (with respect to the other VDE values presented in Table 2) are likely caused by the small electron affinities of OH (1.828 eV), CH_3_ (0.080 eV) and NH_2_ system (0.771 eV). However, the electronic stabilities of these three anions seem surprisingly large in the context of the very small electron affinities of ligands OH, CH_3_ and NH_2_. This may be attribute to the forming of the fourth bond by carbon/nitrogen/oxygen atom linking to the Fe atom (see Fig. 2). The large VDE values of the Fe(CN)_4_ and Fe(OH)_4_ anions further give us confidence that they might also be exploited as superhalogen. It is found that the VDE is always higher than the EA. This can be understood by the fact that the latter corresponds to the energy difference between the ground states of neutral and anion. For most cases, these values should be close to each other. However, there are situations where the difference can be very large such as the present Fe(CN)_4_^−^, Fe(BH_4_)_4_^−^ and Fe(Li_2_H_3_)_4_^−^. This happens where the ground state geometries of neutral and anionic clusters are very different.

#### Charge distribution

**Figure 3:**
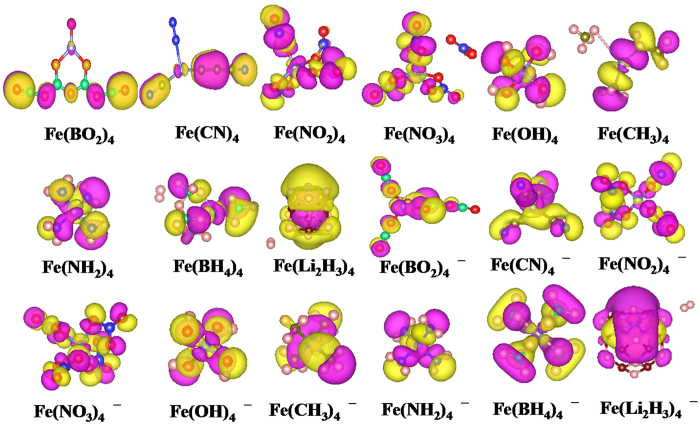
The highest occupied molecular orbitals (HOMOs) of neutral and anionic clusters Fe*L*_4_ (*L* = BO_2_, CN, NO_2_, NO_3_, OH, CH_3_, NH_2_, BH_4_ and Li_2_H_3_).

In order to fully understand the superhalogen properties, the distribution of the extra electronic charge as one moves from neutral to anion are evaluated by the natural population analysis (NPA). The charges on each atom of neutral and anionic Fe*L*_4_ (*L* = BO_2_, CN, NO_2_, NO_3_, OH, CH_3_, NH_2_, BH_4_ and Li_2_H_3_) clusters are given in Figures S3 and S4. We can find that the charge on the Fe atom in all the studied clusters is positive except for the neutral and anionic Fe(Li_2_H_3_)_4_ clusters, resulting from the anticipated Fe to highly electronegative ligands charge transfer. The total charge on the Fe atom in anions Fe*L*_4_^−^ (*L* = BO_2_, NO_2_, NO_3_, and OH) are almost the same as those of their neutral counterparts (see Figures S3 and S4). This obviously indicates that the extra electron in these anions is localized over the ligands, which should contribute considerably to their high EAs. These charge distributions are consistent with their superhalogen characteristics. While this phenomenon also exists in Fe(CH_3_)_4_ and Fe(NH_2_)_4_ clusters, but their EA values are anomalously small compared with other EAs presented in Table 2. This may be attributed to their very small electron affinities of CH_3_ and NH_2_ as mentioned above. As for Fe(CN)_4_ and Fe(BH_4_)_4_, the extra electron is aggregated mainly into ligands CN (0.595e) and BH_4_ (0.736e), respectively. But, there is also non-negligible distribution of the extra electron on the central Fe atom, which is the reason for their low EA values. It is well known that the delocalization of the extra electron can be seen in the highest occupied molecular orbital (HOMO) of cluster. Meanwhile, the HOMO can reveal the bonding nature of cluster. Therefore, we carried out the HOMOs of the studied clusters, and the results for neutral and anionic species are presented in Table S1 and Fig. 3. Generally speaking, the EAs of superhalogen anions are large if their HOMOs belong to non-bonding orbital. Namely, the density of HOMOs of superhalogen anions almost comes from the orbitals of ligands. Thus, we here focus our attention on the HOMOs of anions. From Fig. 3, we clearly find that the interactions between the different constituent ligands, as well as the ligand and central Fe atom are all non-negligible in Fe(CN)_4_^−^, Fe(OH)_4_^−^, Fe(CH_3_)_4_^−^and Fe(NH_2_)_4_^−^ anions. These results in non-bonding and anti-bonding natures of the HOMO coexist in these anions. The non-bonding can cause the repulsion of ligand and the delocalization of electron, which may affect the EA values of superhalogens. The HOMO of Fe(BO_2_)_4_^−^ anion possesses the interaction between ligand and Fe atom and that of the Fe(Li_2_H_3_)_4_^−^ comes from the hydrogen atoms and Fe atom, whereas those of Fe(NO_2_)_4_^−^ and Fe(BH_4_)_4_^−^ occur between their respective ligands. As for Fe(NO_3_)_4_^−^, the HOMO has non-bonding property, which is responsible for its largest EA (6.303 eV) among all the studied systems. By the comparison between the HOMOs of Fe(OH)_4_^−^and Fe(BH_4_)_4_^−^, we conjectured that the difference of their VDE values may be related to the HOMO pattern of their ligands, such as σ-symmetry or π-symmetry.

### Magnetic property

Magnetism is one of the most prominent properties of transition metals. Fe atom, with electronic configuration of 3d^6^4s^2^, carries magnetic moment of 4 μ_B_/atom, and it is coupled ferromagnetically in bulk. It will be, thus, interesting to see if the Fe atom continues to possess magnetic property in our studied Fe*L*_4_ (*L* = BO_2_, CN, NO_2_, NO_3_, OH, CH_3_, NH_2_, BH_4_ and Li_2_H_3_) clusters, and if so whether its values decrease or enhanced with ligand decoration? Because contribution of orbital magnetic moment is usually very small compared with spin magnetic moments in clusters, the spin magnetic moment is though to be reasonable estimate of the total magnetic moment for a given metal-containing cluster. That is to say, the spin magnetic moment is equal to spin multiplicity minus one. In Table 3, we summarized the magnetic moments of neutral and anionic Fe*L*_4_ (L = BO_2_, CN, NO_2_, NO_3_, OH, CH_3_, NH_2_, BH_4_ and Li_2_H_3_) clusters and the local magnetic moments on Fe atom. Meanwhile, the corresponding α and β spin electron configurations of Fe atom are also listed in Table 3. Clearly, it is found that the total magnetic moments of anionic Fe*L*_4_ clusters are always larger than their corresponding neutral counterparts. The anionic clusters, except for Fe(Li_2_H_3_)_4_^−^, carry a magnetic moment of 5 μ_B_/atom, which is consistently 1 μB more than those of neutrals. While in Fe(Li_2_H_3_)_4_, the neutral and anionic isomers possess magnetic moments of 2 μ_B_/atom and 3 μ_B_/atom, respectively. By observing the local spin magnetic moment of Fe atom in these clusters, we note that the main contributions to the total magnetic moments come from 3d orbital of Fe atom. Due to s-p-d hybridization, the 4s and 4p orbitals are slightly polarized, resulting in large total magnetic moments on Fe sites. In conclusion, the Fe atom continues to carry large magnetic moment in our studied systems. Its magnetic moment is almost maintained when Fe atom is decorated with ligands except for neutral and anionic Fe(Li_2_H_3_)_4_.

**Figure 4:**
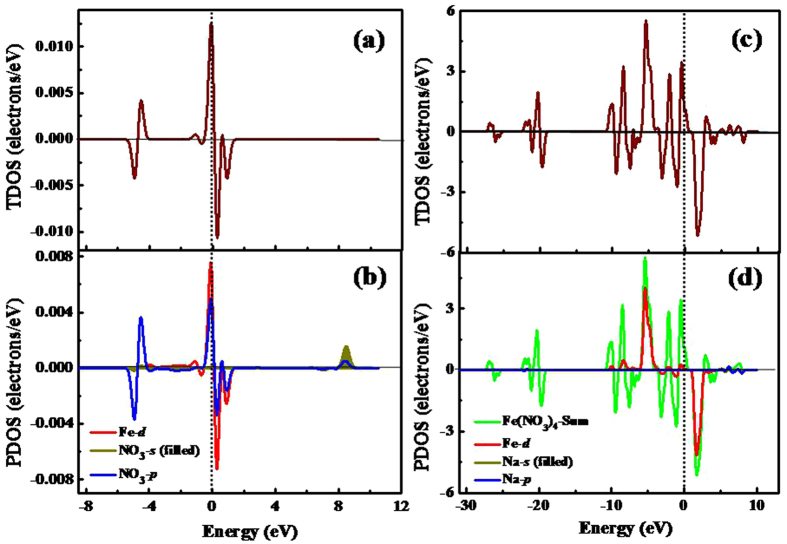
Calculated total spin DOS and partial spin DOS of Fe(NO_3_)_4_ (**a**,**b**), as well as NaFe(NO_3_)_4_ (**c**,**d**). The Fermi level is indicated by the vertical dashed line.

In order to further explore the origin of the magnetic behavior, we calculated the total spin density of states (TDOS) and partial spin density of states (PDOS). Here, we only take the superhalogen Fe(NO_3_)_4_, which possesses the largest EA value among all the studied clusters and the large magnetic moment, as an example. In Fig. 4, the spin density of states of Fe(NO_3_)_4_ and its corresponding hypersalt are presented in left and right columns, respectively. In addition, the spin DOS of the other superhalogens and hypersalts are shown in Figures S5 and S6 of Supplementary Information. The comparison between them may help us to see if the iron atom maintains its ferromagnetism. Overall, the total spin DOS show spin polarization near the Fermi energy. By comparing the total and partial spin DOS, it is obviously found that the total magnetic moments mainly come from the d states of central metal Fe atom, while the contribution of s states of ligands is nearly negligible. This result is in agreement with the above finding of local magnetic moments on Fe atom.

### Hypersalts: NaFeL_4_ (L = BO_2_, CN, NO_2_, NO_3_, OH and BH_4_)

**Figure 5:**
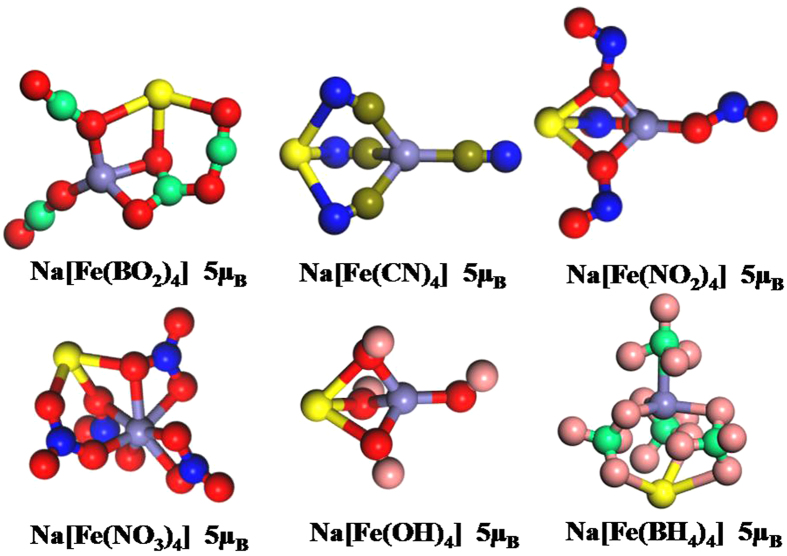
The ground-state structures of (from left) the formed hypersalts Na[Fe(BO_2_)_4_], Na[Fe(CN)_4_], Na[Fe(NO_2_)_4_], Na[Fe(NO_3_)_4_], Na[Fe(OH)_4_] and Na[Fe(BH_4_)_4_] along with their magnetic moments. The gray, red, springgreen, royalblue, blue, olive, pink and maroon spheres represent the Na, O, B, Fe, N, C, H and Li atoms, respectively.

It has been found that Fe(BO_2_)_4_, Fe(NO_3_)_4_ and Fe(NO_2_)_4_ are magnetic superhalogens. Considering the B3LYP may underestimate the electron affinity, Fe(CN)_4_ and Fe(OH)_4_ might also be exploited as magnetic superhalogens. In addition, anion Fe(BH_4_)_4_^−^ has large vertical detachment energy which is comparable to that of Fe(NO_2_)_4_. As is well known, alkali metal-superhalogen complexes (named as hypersalts) can well maintain the independence nature of superhalogen groups, and high binding energies and structural stabilities. More importantly, those complexes have low excitation energy and high polarization rate. These make them play an important role in nonlinear self assembling materials in the future. Therefore, it is very interesting to see if adding a counteraction such as Na^+^ ion to these negative ions can form hypersalts. To obtain the optimized structures of these salt, we added a Na^+^ ion to various possible sites of the ground-state structures and low-lying isomers of superhalogen anions. The optimized ground-state structures of these salt moieties together with their magnetic moments are presented in Fig. 5. As can be seen from Fig. 5, we noted that the structures of ligands Fe*L*_4_ (*L* = BO_2_, CN, NO_2_, NO_3_, OH and BH_4_) in NaFeL_4_ salts are slightly changed compared with its isolated form. The magnetic moments of these salt moieties remain unchanged compared with its ligand anions. This result is further confirmed by comparing the total and partial spin density of states of salt moieties Na[Fe(CN)_4_], Na[Fe(NO_2_)_4_] and Na[Fe(NO_3_)_4_], as illustrated in Figure S6(c,d). In addition, we also found that the magnetic moments of these salt moieties mainly come from Fe-d states, while the magnetic moments of Na-s and Na-p states are nearly negligible, indicating that the spin polarization mainly located on their ligand superhalogen anions.

The thermodynamic stabilities of these salt moieties are estimated from the fragmentation energies computed for different decay channels. Their dissociation energies are calculated as the differences in the total energies of parent and daughters. Here, we consider the two most probable fragmentation channels NaFe*L*_4_ → Na + Fe*L*_4_ and NaFe*L*_4_ → Na^+^ + Fe*L*_4_^−^. Their dissociation energies are obtained using the following formulas:


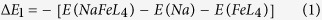



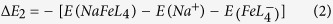


where *E(M*) stand for the total energies of corresponding atoms or clusters. The calculated results are tabulated in Table 4. In principle, positive fragmentation energy indicates that the salts’ cluster is stable against the corresponding fragmentation channel, and vice verse. From Table 4, it is clearly seen that all these salt moieties are thermodynamically stable against dissociation into Na + Fe*L*_4_, whereas the dissociation into Na^+^ + Fe*L*_4_^−^ is less favorable except for Na[Fe(OH)_4_] → Na^+^ + Fe(OH)_4_^−^. More strikingly, in the case of Na[Fe(OH)_4_], the fragmentation channel with Na^+^ loss is the most favorable dissociation.

## Conclusions

In summary, we have carried out a systematic study of the equilibrium geometries, superhalogen properties and magnetic properties of the neutral and anionic Fe*L*_4_ (*L* = BO_2_, CN, NO_2_, NO_3_, OH, CH_3_, NH_2_, BH_4_ and Li_2_H_3_) clusters. It is found that Fe(BO_2_)_4_, Fe(NO_2_)_4_ and Fe(NO_3_)_4_ are the new class of the magnetic superhalogens, and their electron affinities are higher than those of the constituent superhalogens BO_2_, NO_2_ and NO_3_. The extra electron in these complexes is mainly localized over the ligands in anions Fe*L*_4_^−^ (*L* = BO_2_, NO_2_ and NO_3_), which is consistent with their superhalogen characteristics. In addition, the hypersalts consisted of magnetic superhalogen anions and Na^+^ ion are also studied. The results show that the magnetic moments of the formed salts remain unchanged compared with their constituent ligands. The dissociation channel Na + Fe*L*_4_ is the most favorable for all the salts except for Na[Fe(OH)_4_]. This new discovery provides a clue for designing the magnetic hyperhalogens or superhalogens. We hope that our study will stimulate experimental efforts.

## Additional Information

**How to cite this article**: Ping Ding, L. *et al*. Iron-based magnetic superhalogens with pseudohalogens as ligands: An unbiased structure search. *Sci. Rep.* **7**, 45149; doi: 10.1038/srep45149 (2017).

**Publisher's note:** Springer Nature remains neutral with regard to jurisdictional claims in published maps and institutional affiliations.

## Supplementary Material

Supplementary Information

## Figures and Tables

**Table 1:** Electron affinities (eV) of BO_2_, CH_3_, Li_2_H_3_, NH_2_, BH_4_, CN, OH, NO_3_ and NO_2_.

Clusters	B3LYP	CCSD		Exp.
	6-311+G*	6-311+G(2df)	6-311+G*	
BO_2_	4.353	4.326	4.146	4.46^a^
CH_3_	−0.003	0.025	−0.525	0.080^b^
Li_2_H_3_	3.292	3.289	3.241	
NH_2_	0.597	0.622	0.038	0.771^c^
BH_4_	3.287	3.254	2.998	
CN	4.072	4.055	3.627	3.82^d^
OH	1.721	1.712	1.145	1.828^e^
NO_3_	4.034	3.979	4.035	3.95^f^
NO_2_	2.294	2.219	2.038	2.275^g^

^a^Ref. 46. ^b^Ref. 47. ^c^Ref. 48. ^d^Ref. 49. ^e^Ref. 50. ^f^Ref. 51. ^g^Ref. 52.

**Table 2:** Theoretical values of electron affinity (EA), adiabatic detachment energy (ADE) and vertical detachment energy (VDE) of anionic clusters Fe*L*_4_ (*L* = BO_2_, CN, NO_2_, NO_3_, OH, NH_2_, CH_3_, BH_4_ and Li_2_H_3_) at B3LYP and CCSD(T) levels of theory, and the theoretical and experimental values of FeF_4_, FeCl_4_ and FeBr_4_ clusters as comparison.

clusters	B3LYP			CCSD(T)		
	EA	ADE	VDE	EA	ADE	VDE
Fe(BO_2_)_4_^−^	5.410	6.371	6.974	5.469	7.144	8.218
Fe(CN)_4_^−^	3.504	6.421	7.377	3.532	7.327	8.288
Fe(NO_2_)_4_^−^	4.979	5.216	5.823	5.251	5.727	6.953
Fe(NO_3_)_4_^−^	6.303	6.738	6.835	6.459	7.543	7.646
Fe(OH)_4_^−^	3.331	3.331	4.147	4.248	4.248	5.058
Fe(CH_3_)_4_^−^	0.799	1.842	2.731	1.108	2.151	3.004
Fe(NH_2_)_4_^−^	2.068	2.354	3.191	2.842	2.398	3.430
Fe(BH_4_)_4_^−^	2.387	5.305	5.932	1.929	5.824	6.642
Fe(Li_2_H_3_)_4_^−^	0.297	1.169	2.154	0.432	1.204	2.413
FeF_4_^−^	6.091^a^		7.087^a^			
FeCl_4_^−^	6.00 ± 0.08^b^		6.32 ± 0.08^b^			
FeBr_4_^−^	5.50 ± 0.08^b^		5.85 ± 0.08^b^			

All the energies are given in eV. ^a^Ref. 29. ^b^Ref. 57.

**Table 3:** The spin total magnetic moments of neutral and anionic clusters Fe*L*_4_ (*L* = BO_2_, CN, NO_2_, NO_3_, OH, CH_3_, NH_2_, BH_4_ and Li_2_H_3_), as well as the local magnetic moments and the corresponding α and β spin electron configurations on Fe atom inside these clusters.

Clusters	Neutral						Anion	
	μ (μ_B_)		Natural Electron Configuration		μ (μ_B_)		Natural Electron Configuration	
	T	L	α	β	T	L	α	β
Fe(BO_2_)_4_	4	3.52	4s^0.15^3d^4.72^4p^0.19^4d^0.01^	4s^0.07^3d^1.30^4p^0.17^4d^0.01^	5	4.02	4s^0.10^3d^4.95^4p^0.18^4d^0.01^	4s^0.10^3d^0.94^4p^0.17^4d^0.01^
Fe(CN)_4_	4	3.80	4s^0.28^3d^4.91^4p^0.17^4d^0.01^	4s^0.14^3d^1.29^4p^0.14^	5	4.03	4s^0.26^3d^4.91^4p^0.51^	4s^0.25^3d^1.08^4p^0.33^
Fe(NO_2_)_4_	4	3.94	4s^0.16^3d^4.93^4p^0.22^4d^0.01^	4s^0.16^3d^0.99^4p^0.22^4d^0.01^	5	4.05	4s^0.14^3d^4.94^4p^0.22^4d^0.01^	4s^0.13^3d^0.92^4p^0.20^4d^0.01^
Fe(NO_3_)_4_	4	3.92	4s^0.15^3d^4.94^4p^0.24^4d^0.02^	4s^0.15^3d^0.90^4p^0.25^4d^0.01^	5	4.06	4s^0.16^3d^4.92^4p^0.29^4d^0.03^	4s^0.16^3d^1.01^4p^0.29^4d^0.02^
Fe(OH)_4_	4	3.18	4s^0.14^3d^4.57^4p^0.23^4d^0.01^	4s^0.11^3d^1.42^4p^0.24^4d^0.01^	5	4.05	4s^0.12^3d^4.97^4p^0.22^4d^0.01^	4s^0.11^3d^0.94^4p^0.21^4d^0.01^
Fe(CH_3_)_4_	4	3.44	4s^0.24^3d^4.75^4p^0.24^4d^0.01^	4s^0.20^3d^1.41^4p^0.18^4d^0.01^	5	4.01	4s^0.23^3d^4.95^4p^0.31^4d^0.01^	4s^0.21^3d^1.08^4p^0.19^4d^0.01^
Fe(NH_2_)_4_	4	2.88	3d^4.56^4p^0.28^5s^0.16^4d^0.02^	3d^1.72^4p^0.29^5s^0.14^4d^0.01^	5	3.88	4s^0.15^3d^4.95^4p^0.28^4d^0.01^	4s^0.15^3d^1.09^4p^0.26^4d^0.01^
Fe(BH_4_)_4_	4	3.37	4s^0.11^3d^4.92^4p^0.26^4d^0.03^	4s^0.11^3d^1.57^4p^0.26^4d^0.01^	5	3.40	4s^0.16^3d^4.87^4p^0.39^4d^0.04^	4s^0.15^3d^1.51^4p^0.40^4d^0.01^
Fe(Li_2_H_3_)_4_	2	1.96	4s^0.22^3d^4.54^4p^0.57^4d^0.02^	4s^0.19^3d^2.68^4p^0.50^4d^0.02^	3	2.83	3d^4.90^4p^0.41^5s^0.20^4d^0.01^	3d^2.23^4p^0.28^5s^0.17^4d^0.01^

**Table 4:** The fragment channels and dissociation energies (eV) of the salt moieties formed by adding a Na^+^ ion on superhalogen anions Fe(BO_2_)_4_, Fe(CN)_4_, Fe(NO_2_)_4_, Fe(NO_3_)_4_, Fe(OH)_4_ and Fe(BH_4_).

Cluster	Channel	Δ*E*_1_	Channel	Δ*E*_2_
Na[Fe(BO_2_)_4_]	Na[Fe(BO_2_)_4_] → Na + Fe(BO_2_)_4_	9.598	Na[Fe(BO_2_)_4_] → Na^+^ + Fe(BO_2_)_4_^−^	−1.232
Na[Fe(CN)_4_]	Na[Fe(CN)_4_] → Na + Fe(CN)_4_	7.890	Na[Fe(CN)_4_] → Na^+^ + Fe(CN)_4_^−^	−1.117
Na[Fe(NO_2_)_4_]	Na[Fe(NO_2_)_4_] → Na + Fe(NO_2_)_4_	9.714	Na[Fe(NO_2_)_4_] → Na^+^ + Fe(NO_2_)_4_^−^	−0.686
Na[Fe(NO_3_)_4_]	Na[Fe(NO_3_)_4_] → Na + Fe(NO_3_)_4_	11.047	Na[Fe(NO_3_)_4_] → Na^+^ + Fe(NO_3_)_4_^−^	−0.677
Na[Fe(OH)_4_]	Na[Fe(OH)_4_] → Na + Fe(OH)_4_	9.504	Na[Fe(OH)_4_] → Na^+^ + Fe(OH)_4_^−^	0.751
Na[Fe(BH_4_)_4_]	Na[Fe(BH_4_)_4_] → Na + Fe(BH_4_)_4_	6.883	Na[Fe(BH_4_)_4_] → Na^+^ + Fe(BH_4_)_4_^−^	−0.925
