# Supplementary material for: Iron-based magnetic superhalogens with pseudohalogens as ligands: An unbiased structure search
Source: Sci Rep. 2017 Mar 22;7:45149. doi: 10.1038/srep45149 (PMC5361091; doi:10.1038/srep45149)
Supplement: Supplementary Information [file srep45149-s1.pdf]

# Iron-based magnetic superhalogens with pseudohalogens as ligands: An unbiased structure search

Li Ping Ding[1], Peng Shao[2,*], Cheng Lu[3,*], Fang Hui Zhang[1], Li Ya Wang[3,*]

[1]Department of Optoelectronic Science & Technology, College of Elecrical & Information Engineering, Shanxi University of Science & Technology, Xian, 710021, China.

[2]Department of Physics, Shaanxi University of Science & Technology, Xi'an, 710021, China.

[3]Department of Physics, Nanyang Normal University, Nanyang, 473061, China.

*Correspondence author. E-mail: scu_sp@163.com (Peng Shao), lucheng@calypso.cn (Cheng Lu), wly@nynu.edu.cn (Li Ya Wang).

| Neutral | | | Anion | | |
|---|---|---|---|---|---|
| Isomer | distance (Å) | frequency ($cm^{-1}$) | Isomer | distance (Å) | frequency ($cm^{-1}$) |
| $Fe(BO_2)_4$ | 1.649 | 2097, 2080,1397, 1365, 1344, 1271 | $Fe(BO_2)_4^-$ | 1.878 | 2061, 2035, 2015, 1334, 1258, 1163 |
| $Fe(CN)_4$ | 1.954 | 2456, 2330, 2227, 2130, 981, 573 | $Fe(CN)_4^-$ | 2.021 | 2210, 2209, 2209, 2208, 428, 427 |
| $Fe(NO_2)_4$ | 1.834 | 1750, 1724, 1719, 1350, 1333, 1221 | $Fe(NO_2)_4^-$ | 1.905 | 1631, 1612, 1611, 1611, 954, 918 |
| $Fe(NO_3)_4$ | 2.060 | 1695, 1673, 1672, 1198, 1186, 1183 | $Fe(NO_3)_4^-$ | 2.097 | 1610, 1585, 1578, 1578, 1283, 1275 |
| $Fe(OH)_4$ | 1.787 | 3776, 3772, 3772, 3771, 845, 835 | $Fe(OH)_4^-$ | 1.899 | 3807, 3807, 3806, 3806, 679, 678 |
| $Fe(CH_3)_4$ | 2.036 | 3132, 3128, 3127, 3066, 3065, 3041 | $Fe(CH_3)_4^-$ | 2.114 | 3027, 3026, 3026, 3024, 3023, 3023 |
| $Fe(NH_2)_4$ | 1.847 | 3645, 3644, 3630, 3629, 3536, 3532 | $Fe(NH_2)_4^-$ | 1.972 | 3563, 3560, 3559, 3558, 3468, 3466 |
| $Fe(BH_4)_4$ | 1.874 | 4393, 2646, 2641, 2622, 2618, 2557 | $Fe(BH_4)_4^-$ | 1.859 | 2534, 2533, 2533, 2528, 2482, 2473 |
| $Fe(Li_2H_3)_4$ | 1.669 | 4348, 1495, 1399, 1383, 1337, 1282 | $Fe(Li_2H_3)_4^-$ | 1.756 | 4321, 1410, 1366, 1198, 1147, 1138 |

**Table S1.** The structure information, including the shortest distance between central Fe and ligand atoms, and the vibration frequencies with most IR intensities of the lowest-energy structures for neutral and anionic $FeL_4$ ($L$ = $BO_2$, CN, $NO_2$, $NO_3$, OH, $CH_3$, $NH_2$, $BH_4$ and $Li_2H_3$) clusters.

| Isomer | state | HOMO (eV) | LUMO (eV) | HL (eV) | Isomer | state | HOMO (eV) | LUMO (eV) | HL (eV) |
|---|---|---|---|---|---|---|---|---|---|
| $Fe(BO_2)_4$ | $^5B_2$ | -9.531 | -6.348 | 3.183 | $Fe(BO_2)_4^-$ | $^6A$ | -4.884 | -1.072 | 3.812 |
| $Fe(CN)_4$ | $^5A$ | -7.936 | -5.185 | 2.751 | $Fe(CN)_4^-$ | $^6A$ | -5.573 | -1.876 | 3.697 |
| $Fe(NO_2)_4$ | $^5A$ | -8.211 | -5.769 | 2.442 | $Fe(NO_2)_4^-$ | $^6A$ | -4.175 | -1.041 | 3.135 |
| $Fe(NO_3)_4$ | $^5A$ | -9.399 | -7.093 | 2.306 | $Fe(NO_3)_4^-$ | $^6A$ | -5.133 | -1.430 | 3.703 |
| $Fe(OH)_4$ | $^5A$ | -8.731 | -4.381 | 4.350 | $Fe(OH)_4^-$ | $^6A$ | -2.008 | 2.391 | 4.399 |
| $Fe(CH_3)_4$ | $^5A$ | -6.081 | -2.877 | 3.204 | $Fe(CH_3)_4^-$ | $^6A$ | -0.976 | 2.424 | 3.400 |
| $Fe(NH_2)_4$ | $^5A$ | -6.404 | -3.109 | 3.386 | $Fe(NH_2)_4^-$ | $^6A$ | -0.959 | 2.486 | 3.446 |
| $Fe(BH_4)_4$ | $^5A$ | -7.742 | -3.908 | 3.835 | $Fe(BH_4)_4^-$ | $^6A$ | -4.238 | -0.702 | 3.536 |
| $Fe(Li_2H_3)_4$ | $^3A$ | -4.758 | -1.233 | 3.524 | $Fe(Li_2H_3)_4^-$ | $^4A$ | -0.697 | 1.440 | 2.137 |

**Table S2.** Electronic state, highest occupied molecular orbital (HOMO), lowest unoccupied molecular orbital (LUMO) and HOMO-LUMO gaps (HL) of the lowest-energy structures for neutral and anionic $FeL_4$ ($L$ = $BO_2$, CN, $NO_2$, $NO_3$, OH, $CH_3$, $NH_2$, $BH_4$ and $Li_2H_3$) clusters.

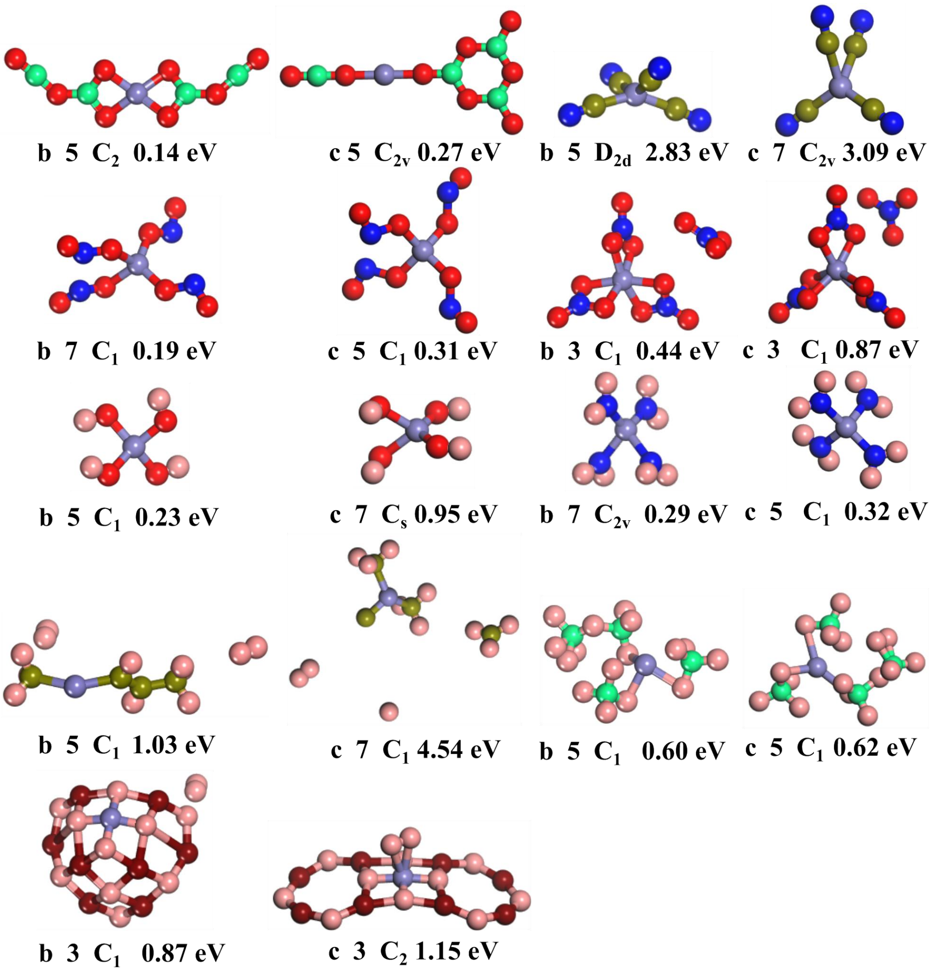


**Figure S1.** The low-lying isomers of neutral Fe*L*$_4$ (*L* = $BO_2$, CN, $NO_2$, $NO_3$, OH, $NH_2$, $CH_3$, $BH_4$ and $Li_2H_3$) clusters along with their spin multiplicities, symmetries and relative energies.

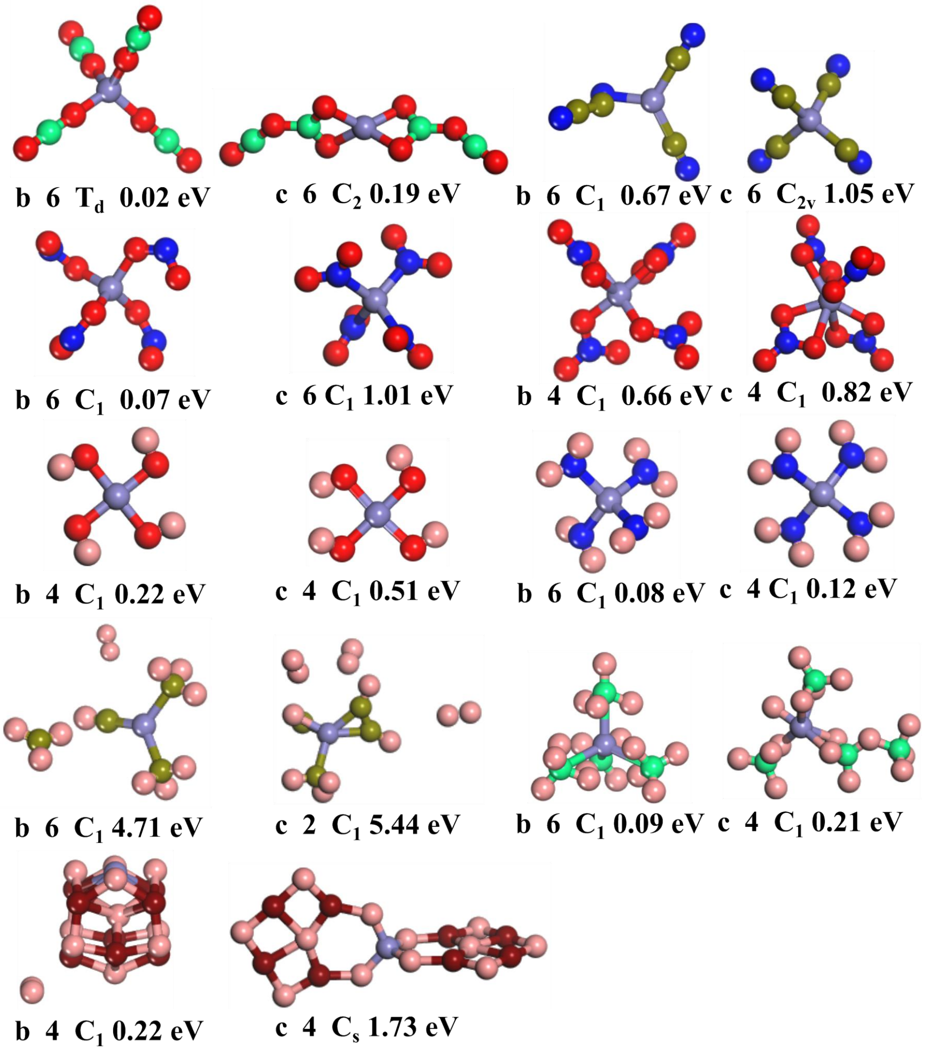


**Figure S2.** The low-lying isomers of anionic Fe$L_4$ ($L$ = $BO_2$, CN, $NO_2$, $NO_3$, OH, $NH_2$, $CH_3$, $BH_4$ and $Li_2H_3$) clusters along with their spin multiplicities, symmetries and relative energies.

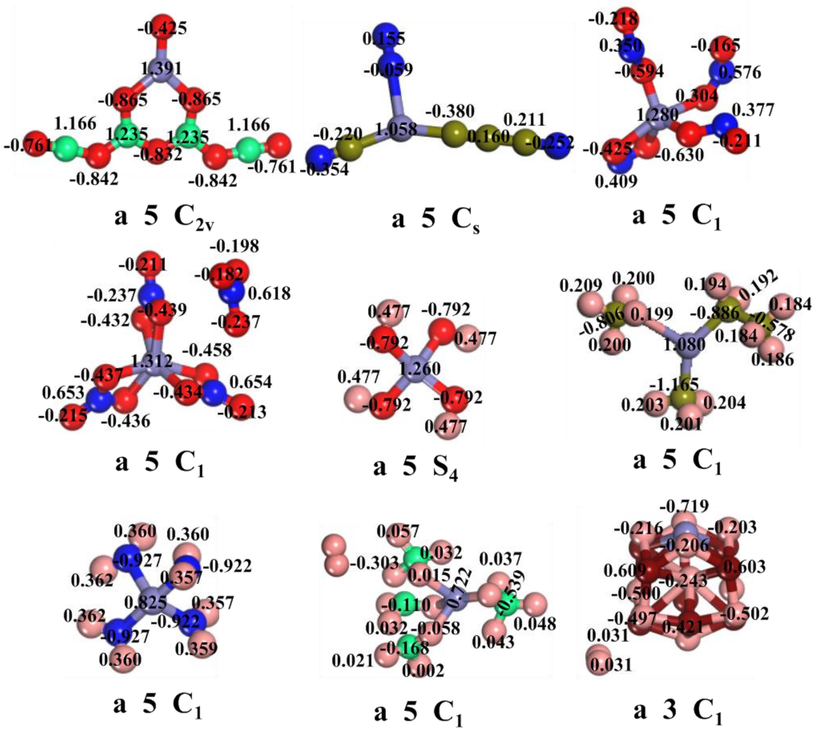


**Figure S3.** The ground-state structures of neutral clusters Fe$L_4$ ($L$ = $BO_2$, CN, $NO_2$, $NO_3$, OH, $CH_3$, $NH_2$, $BH_4$ and $Li_2H_3$) along with their spin multiplicities and symmetries. The numbers in structures are the NPA charges on each atom. The red, springgreen, royalblue, blue, olive, pink and maroon spheres represent the O, B, Fe, N, C, H and Li atoms, respectively.

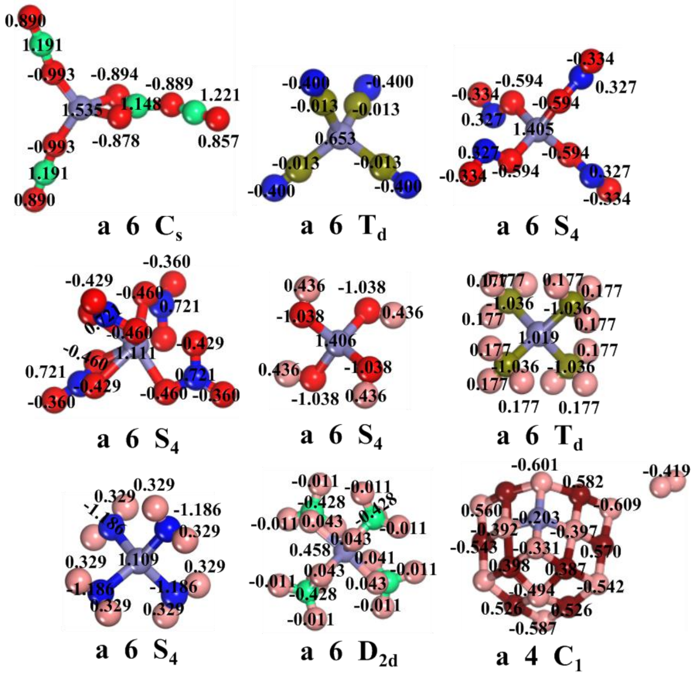


**Figure S4.** The ground-state structures of anionic clusters Fe$L_4$ ($L$ = $BO_2$, CN, $NO_2$, $NO_3$, OH, $CH_3$, $NH_2$, $BH_4$ and $Li_2H_3$) along with their spin multiplicities and symmetries. The numbers in structures are the NPA charges on each atom. The red, springgreen, royalblue, blue, olive, pink and maroon spheres represent the O, B, Fe, N, C, H and Li atoms, respectively.

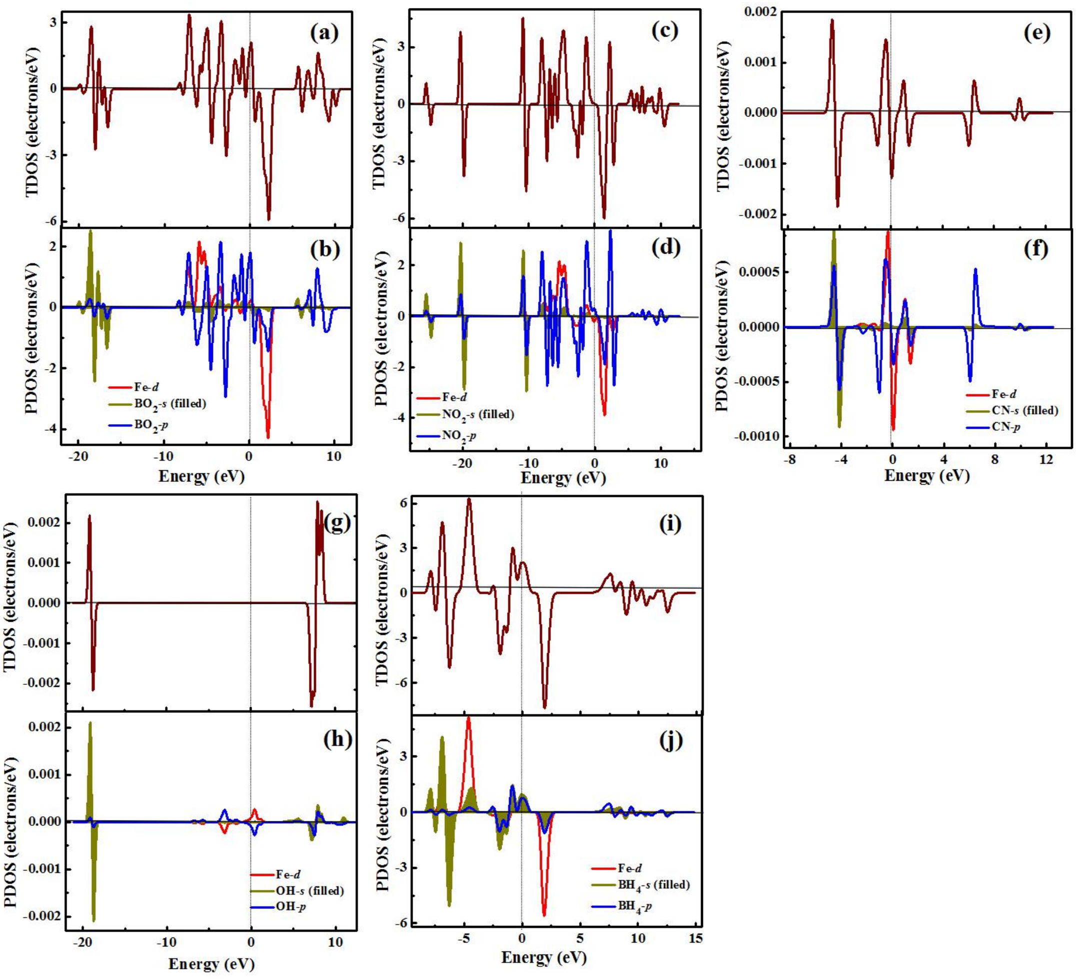


**Figure S5.** Calculated total spin DOS and partial spin DOS of $Fe(NO_3)_4$ [(a) and (b)], $Fe(NO_2)_4$ [(c) and (d)], $Fe(CN)_4$ [(e) and (f)], $Fe(OH)_4$ [(g) and (h)], as well as $NaFe(BH_4)_4$ [(i) and (j)]. The Fermi level is indicated by the vertical dashed line.

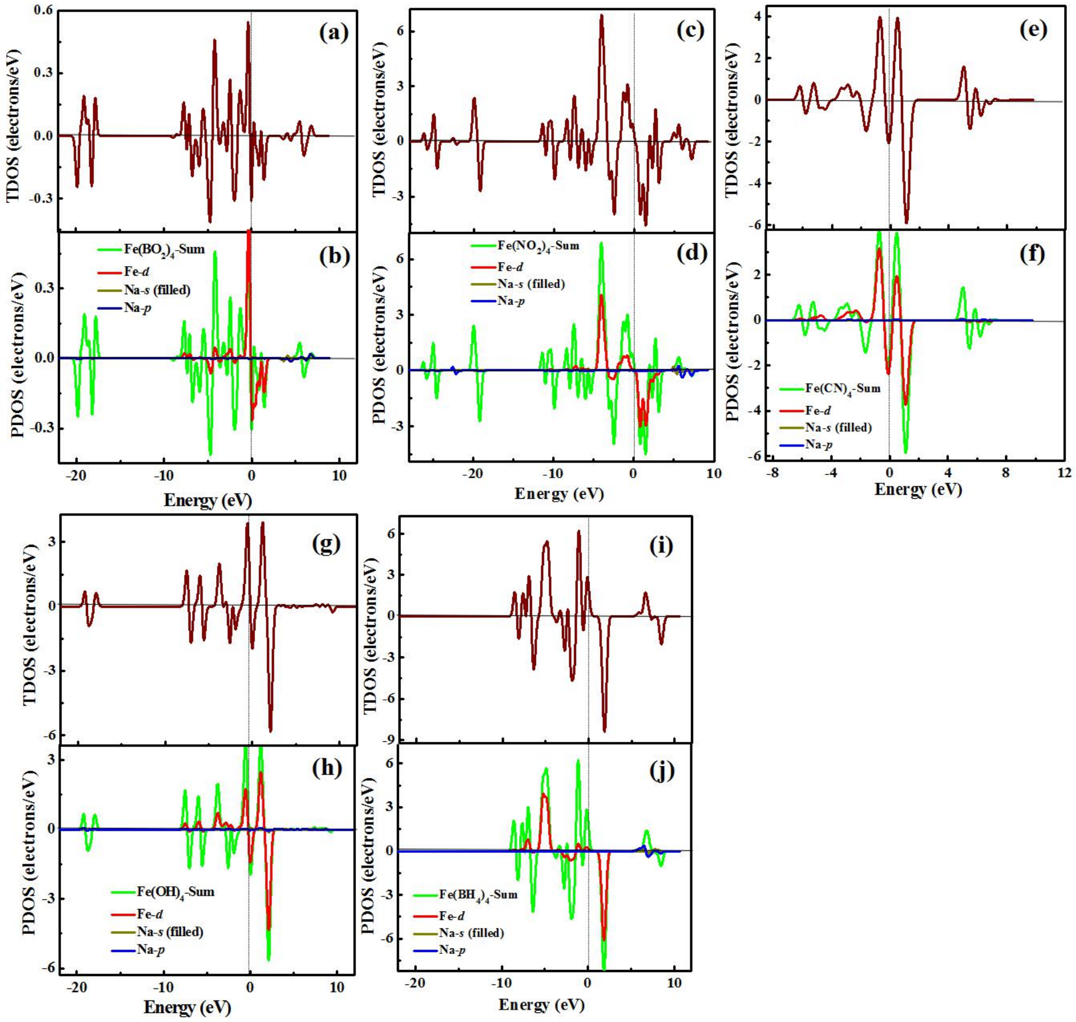


**Figure S6.** Calculated total spin DOS and partial spin DOS of hypersalts $NaFe(NO_3)_4$ [(a) and (b)], $NaFe(NO_2)_4$ [(c) and (d)], $NaFe(CN)_4$ [(e) and (f)], $NaFe(OH)_4$ [(g) and (h)], as well as $NaFe(BH_4)_4$ [(i) and (j)]. The Fermi level is indicated by the vertical dashed line.
